# Evidence for Letter-Specific Position Coding Mechanisms

**DOI:** 10.1371/journal.pone.0068460

**Published:** 2013-07-02

**Authors:** Stéphanie Massol, Jon Andoni Duñabeitia, Manuel Carreiras, Jonathan Grainger

**Affiliations:** 1 Basque Center on Cognition, Brain and Language, Donostia, Spain; 2 IKERBASQUE - Basque Foundation for Science, Bilbao, Spain; 3 CNRS and Aix-Marseille University, Marseille, France; McGill University, Canada

## Abstract

The perceptual matching (same-different judgment) paradigm was used to investigate precision in position coding for strings of letters, digits, and symbols. Reference and target stimuli were 6 characters long and could be identical or differ either by transposing two characters or substituting two characters. The distance separating the two characters was manipulated such that they could either be contiguous, separated by one intervening character, or separated by two intervening characters. Effects of type of character and distance were measured in terms of the difference between the transposition and substitution conditions (transposition cost). Error rates revealed that transposition costs were greater for letters than for digits, which in turn were greater than for symbols. Furthermore, letter stimuli showed a gradual decrease in transposition cost as the distance between the letters increased, whereas the only significant difference for digit and symbol stimuli arose between contiguous and non-contiguous changes, with no effect of distance on the non-contiguous changes. The results are taken as further evidence for letter-specific position coding mechanisms.

## Introduction

There is a general consensus nowadays that visual word recognition is essentially letter-based, at least in languages that use an alphabetic script. Within this perspective, efficient reading requires the association of different letter identities with different positions in the printed word, and the question of how letter position information is encoded has become a major issue in reading research in the last decade (see 1 for a review). One key question is whether the mechanism used to code for letter position information in printed words is essentially the same mechanism as might be used to code for positional information in arrays of any kind of visual object. Different approaches to letter position coding provide different answers to this question depending on how they account for the kind of flexibility in position coding that has been revealed by recent research. One phenomenon in particular has been used to illustrate this flexibility – the fact that we can easily read text in which letter odrer has been slightly mofidied. More precisely, an impressive amount of evidence obtained from various paradigms suggests that letter strings formed by transposing two letters of a real word are perceived as being more perceptually similar to the base word than letter strings formed by substituting two letters of the base word [2-9]. As noted by Grainger (2008), these transposed-letter effects have become one of the principle benchmark phenomena that models of orthographic processing must account for.

There are two very different accounts of transposed-letter effects. One account [10,11] proposes that they reflect the operation of generic noise (i.e., object-position uncertainty [12,13] on an otherwise rigid position-coding mechanism. Such models were developed specifically to account for transposed-letter effects by adding positional noise to a position-coding mechanism that cannot otherwise produce transposition effects (i.e., slot-coding [14]. Another class of models [15-18] have proposed letter-specific position coding mechanisms in order to account for location-invariant, and to a certain extent, length-independent orthographic processing [19-21]. It was subsequently discovered that such letter-specific coding mechanisms could also account for transposed-letter effects [22]. According to these models, transposed-letter effects are the result of the very mechanism used to code for letter position information. Of course noise will affect processing in these models, just like it will affect processing of positional information for any kind of visual object (i.e., generic positional noise), but this noise operates on top of a mechanism that is already endowed with a certain amount of positional flexibility.

Models that apply generic positional noise [10,11] predict that letter stimuli should behave like other kinds of visual stimuli, at least when familiarity is controlled for. In support of this approach, García-Orza, Perea and Muñoz (2010) [23] used the masked priming version of the perceptual matching task in order to investigate transposition effects on different types of stimuli (letter strings, digit strings, symbol strings and pseudo-letter strings). Results showed that the transposition priming effects were not specific to letter strings, supporting the hypothesis that position coding takes place before the distinction of different types of stimuli. Critically, a highly similar transposed-character effect was found for letter, digit and symbol strings, suggesting a generic position-coding scheme which is governed by domain-general principles (see also [24].

However, one recent study [25] has provided clear evidence for greater transposition costs for letter stimuli compared with both digit and symbol stimuli. Duñabeitia et al. combined the perceptual matching task with ERP recordings in order to explore changes in character position coding in different types of strings (i.e., letters, digits and symbols). In their experiment, the authors used the classic version of the perceptual matching task [26,27], in which a reference stimulus is explicitly presented, immediately followed by a target stimulus. Participants are then asked to judge whether or not the two stimuli are the same. Duñabeitia et al. observed an early transposed-character similarity effect only for letter strings, while a generalized transposed-character similarity effect arose at around 350ms post-target onset for all types of characters. Furthermore, behavioral data showed that transposition costs (difference between the transposed and substitution conditions) were significantly greater for letter strings compared with the other types of characters. Interestingly, these data highlighted that the most familiar items (which presumably are the letter strings) are the ones that suffer the greatest level of positional uncertainty as compared to other items (i.e., digit and symbol strings), leading to the greatest transposition costs.

The only way that models that apply generic positional noise can account for the greater transposition costs found for letters compared with digits and symbols in the Duñabeitia et al. (2012) study, is by postulating that such noise is greater for letter stimuli. Although this is a possibility, it runs counter to the evidence suggesting that if anything, positional noise should be reduced for letters compared with other kinds of visual stimuli [28]. On the other hand, the greater transposition cost found for letter stimuli in the Duñabeitia et al. study is perfectly in line with models according to which such costs are at least partly driven by flexible letter-specific position coding mechanisms.

Given the theoretical importance of Duñabeitia et al.’s (2012) finding of differential transposition costs for letters and other kinds of visual stimuli, the present study provides a further examination of such effects. Here we go one important step further than the Duñabeitia et al. study by manipulating the distance (measured in number of characters) separating the two transposed elements in strings of letters, digits, and symbols. Participants were presented with pairs of 6-character strings and were asked to decide whether they were identical or different. The two strings could be identical or could differ by transposing or replacing two contiguous characters, two non-contiguous that were 1-character apart, or two non-contiguous characters with two intervening characters.

Prior research has shown that transposed-letter effects can be also obtained with nonword primes involving transpositions of non-contiguous letters (e.g., cholocate-CHOCOLATE, e.g., [5, 7, 29, 30]). The magnitude of the transposed-letter effect highly depends on the number of other letters intervening between the two transposed-letters, diminishing as a function of this distance (e.g., contiguous, 1-letter apart, 2-letter apart; see 5. We therefore expected to observe a diminishing transposition cost as distance increases in the present study. More important, this manipulation of distance provides us with another opportunity for observing a dissociation between letter strings and other kinds of stimuli, which is the focus of the present work. That is, given the hypothesized role of letter-specific position coding mechanisms, letter strings might not only exhibit greater transposition costs than the other kinds of stimuli, but these transposition costs might also be differentially modulated by distance.

## Method

### 1. Ethics Statement

All the participants signed informed consent forms before the experiment and were appropriately informed regarding the basic procedure of the experiment, according to the ethical commitments established by the BCBL Scientific Committee and by the BCBL Ethics Committee that approved the experiment.

### 2. Participants

32 participants (16 women) with a mean age of 22.06 (SD =2.34) years took part in the experiment. They were paid for their collaboration. All of them were native speakers of Spanish and had normal or corrected-to-normal vision.

### 3. Materials

1296 reference-target pairs were used as stimuli. Each of the pairs was composed of two 6-character long strings of uppercase consonants, digits, or meaningful symbols. These three categories were assigned to three blocks, so that each block consisted of 432 letter strings, 432 digit strings, or 432 symbol strings. For the digit strings, the numbers 1, 2, 3, 4, 5, 6, 7, 8 and 9 were used. For the letter strings, the uppercase version of the consonants, G, N, D, K, F, T, S, B and L were used. For the symbol strings, the characters % ? , !, &, +, <, ), $ and # were used. While digit-pair judgments seem to be unaffected by the similarity between the digits and existing letters [31], it is unknown whether or not the same would hold for letter-like symbols. Hence, in order to minimize the potential impact of the £€€T effect [32,33], we decided to substitute two of the letter-like symbols that were used previously in other studies. However, even if the symbols are not exactly matched between studies [25,28], we do not predict any difference in the processing of the symbol strings across experiments, given that in all cases only symbols being highly familiar to the participants were used. The same reference stimulus appeared twice in the experiment, once requiring a “same” response and once a “different” response. In each block, half of the items required a “same” response (216 trials, i.e., 349256-349256, DKLNFT-DKLNFT, & +! ? $ #-& +! ? $ #). The other half (216 trials) required a “different” response. Half of the different pairs differed by means of character transpositions (i.e., transposed condition) or of character replacements (i.e., replaced condition). The distance between the two transposed or replaced characters was also manipulated, measured in terms of number of intervening characters between the two critical ones (i.e., 72 trials per block of contiguous transpositions or replacements, DK LNFT-DL KNFT; 72 trials including non-contiguous transpositions or replacements with one intervening character, KTDLNB-KLDTNB; 72 trials with non-contiguous transpositions or replacements with two intervening characters, LNBKTD-LTBKND). Critically, transpositions or replacements never involved the outer characters. The same proportion of transpositions or replacements was carried out in all the possible within-string locations. Following a counterbalanced design, the reference-target pairs were separated into two subsets to create two lists of experimental stimuli that were presented to different participants.

### 4. Procedure

**Figure 1:**
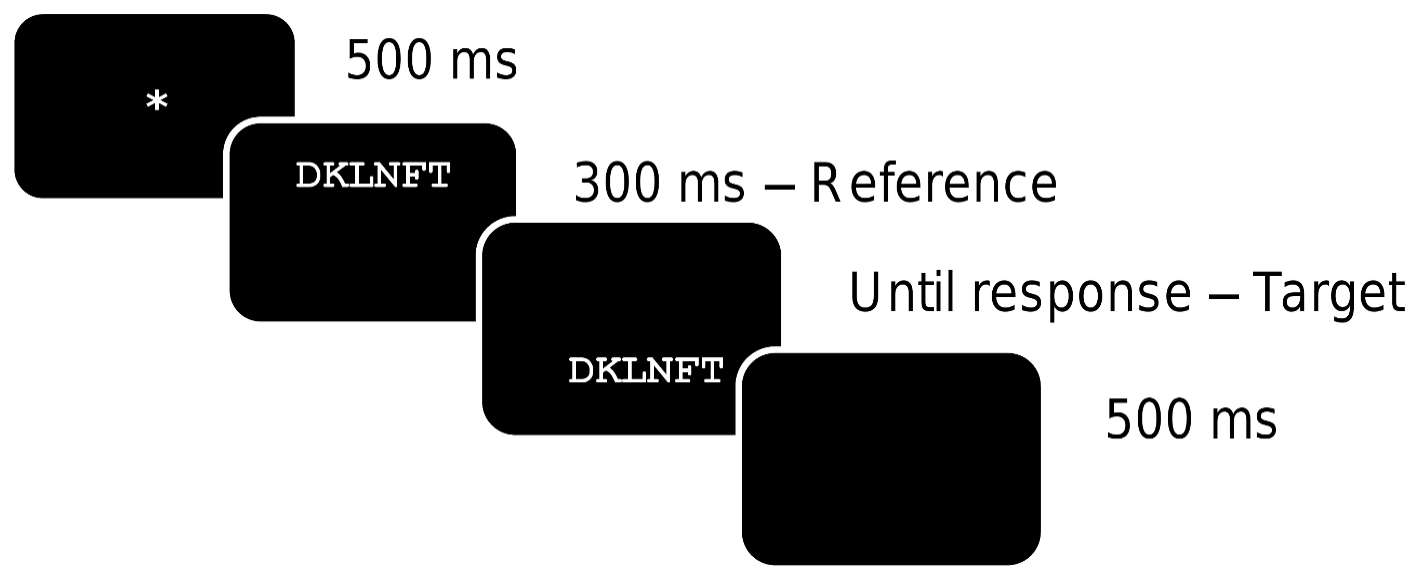
Shematic representation of an experimental trial.

The presentation of the stimuli and recording of the responses were carried out using Presentation software. All stimuli were presented on a CRT monitor. Participants were informed that two strings of characters were going to be subsequently displayed. All stimuli were presented in white Courier New font (size 16 pt.) on a black background. Each trial began with the centered presentation of a fixation stimulus (*) displayed for 500ms. Immediately after this, the reference was presented for 300ms horizontally centered and positioned 3mm above the exact center of the screen. The reference was immediately replaced by the target stimulus that was horizontally centered and positioned 3mm below the center of the screen. Target stimulus remained on the screen for 2000ms or until a response was given. Each trial ended with a blank screen displayed for 500ms. The manipulation of the location of references and targets on the vertical axis was carried out in order to avoid physical overlap between the two strings (see Figure 1 for a schematic representation of a trial). Participants were instructed to decide as rapidly and as accurately as possible whether or not the two strings were exactly identical. They responded “same” by pressing the “L” button on the keyboard and “different” by pressing the “S” button. The experiment was divided in three separate blocks that only included items belonging to the same stimulus category. A short practice session was administered before the main experiment to familiarize participants with the procedure and the task.

## Results

**Figure 2:**
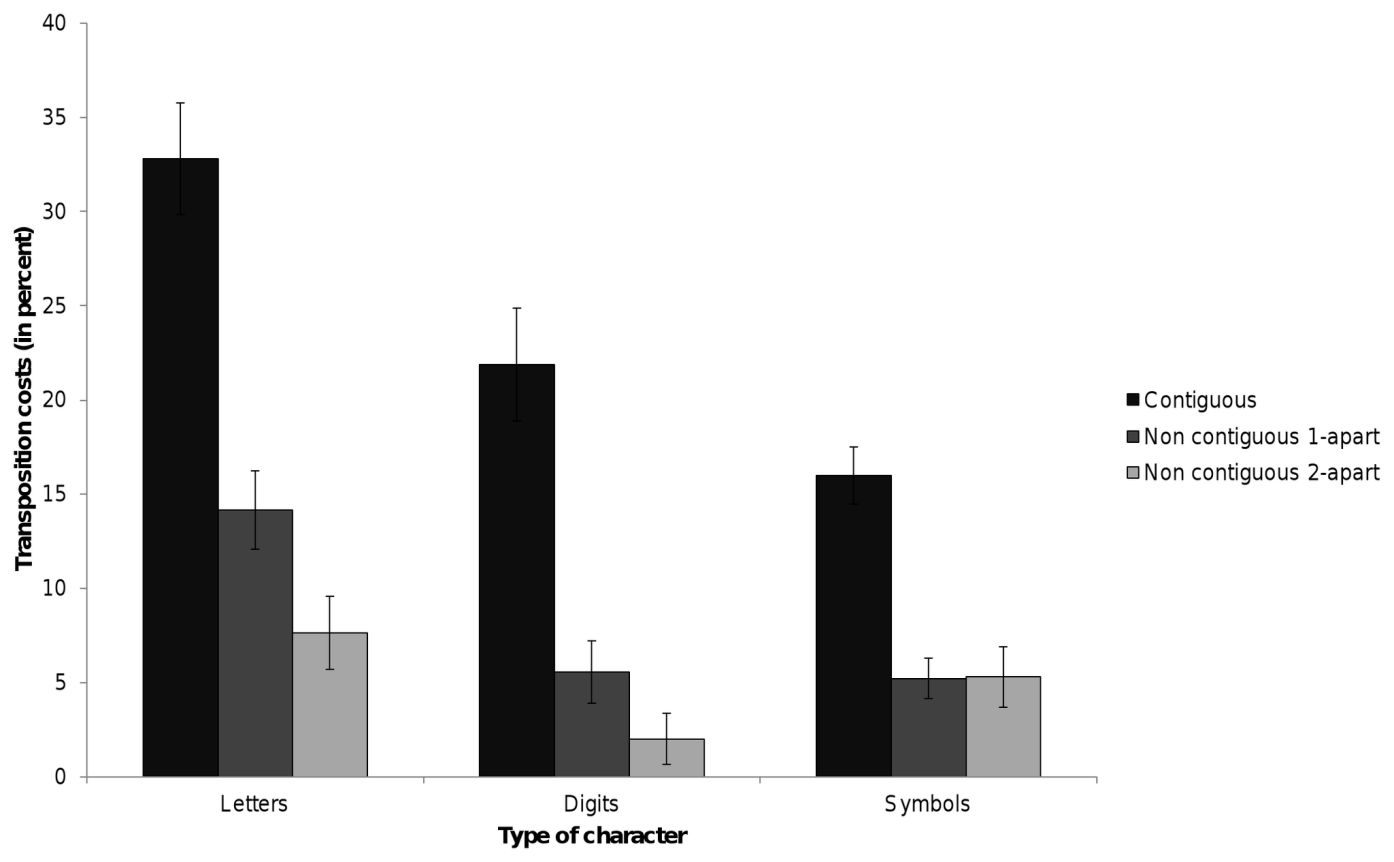
Transposition costs (related – unrelated) in percentage obtained in each experimental condition in the experiment.

Statistical analyses were performed only over the “different” trials, since there was no experimental manipulation within the set of “same” trials. Incorrect responses and reaction times below 250ms and above 1300ms (1.34% of the data) were excluded from the latency analysis. Mean latencies for correct responses and error rates are presented in Table 1. The transposition costs are presented in Figure 2. ANOVAs over participants and items on the transposition costs (the result of the data in the replacement condition minus the data in the transposition condition) on the response latencies and on the error rates were conducted based on a 3 (Type of Character: letter, digit, symbol) x 3 (Distance: contiguous, non-contiguous 1-apart, non-contiguous 2-apart) factorial design.

**Table 1:** Mean reaction times (in ms) and percentage of errors (in italics) for the “different” trials obtained in the Experiment.

		Contiguous			Non contiguous 1-apart			Non contiguous 2-apart		
Type of Characters		Transposition	Replacement	Transposition cost	Transposition	Replacement	Transposition cost	Transposition	Replacement	Transposition cost
**LETTERS**	RT	624.11	605.43	-18.69	621.55	608.58	-12.97	621.66	612.01	-9.65
	*Error*	*52.34*	*19.53*	*-32.81*	*29.43*	*15.28*	*-14.15*	*23.87*	*16.23*	*-7.64*
**DIGITS**	RT	631.56	598.25	-33.31	608.95	595.72	-13.23	608.93	599.85	-9.08
	*Error*	*37.67*	*15.80*	*-21.88*	*15.28*	*9.72*	*-5.56*	*19.70*	*17.71*	*-2.00*
**SYMBOLS**	RT	619.07	596.31	-22.76	594.01	585.23	-8.78	602.03	590.83	-11.20
	*Error*	*34.38*	*18.40*	*-15.97*	*16.41*	*11.20*	*-5.21*	*19.79*	*14.50*	*-5.30*

Note: Mean reaction times and percentage of errors for the “same” trials were 590 ms (7.29%), 596 ms (11.41%) and 601 ms (10.85%) for the digit, letter and symbol strings, respectively; *Transposition cost* is the outcome of the subtraction of the values in the Transposition condition from those in the Replacement condition.

### 1. Reaction times

The latency analyses revealed a significant effect of Distance, F1(2,62) = 3.79, p=.028; F2(2,142) = 4.60, p=.012, showing that the transposition cost was larger for contiguous manipulations than for non-contiguous manipulations. Follow-up analyses revealed that the Distance effect was larger for contiguous than for non-contiguous 1-apart, F1(1,31) = 4.00, p=.054; F2(1,71) = 4.79, p=.032, and for contiguous than 2-apart, F1(1,31) = 5.90, p=.021; F2(1,71) = 8.35, p=.005 (25ms vs. 12ms vs. 10ms, respectively). However the magnitude of the transposition cost for strings that involved manipulations on non-contiguous 1-apart and non-contiguous 2-apart characters did not reach significance (Fs<1). The effect of Type of Character was not significant (Fs<1) and did not interact with the Distance factor (Fs<1).

### 2. Error rates

The error rate analyses showed a significant main effect of Type of Character, F1(2,62) = 14.23, p<.001; F2(2,142) = 24.59, p<.001. A significant main effect of Distance was also observed, F1(2,62) = 75.48, p=<.001; F2(2,142) = 61.86, p<.001. The interaction between the two factors was significant, suggesting that the magnitude of the transposition cost differed across character types and distances, F1(4,124) = 5.53, p=.001; F2(4,284) = 3.82, p=.006. The interaction is illustrated in Figure 2, where we can see the different influence of the distance factor for the three types of stimulus. In the following sections we will unravel this interaction by looking separately at the transposition cost for the different distances (the Distance effect), and at the transposition cost for the different types of characters (the Character effect).

### 2.1 The Distance Effect

Subsequent pairwise comparisons for letter strings showed that the transposition costs in the error rates were significantly different for the three distances: contiguous vs. non-contiguous 1-apart, F1(1,31) = 43.96, p<.001; F2(1,71) = 45.33, p<.001; contiguous vs. non-contiguous 2-apart, F1(1,31) = 84, p<.001; F2(1,71) = 50.35, p<.001, non-contiguous 1-apart vs. non-contiguous 2-apart, F1(1,31) = 6.81, p=.014; F2(1,71) = 4.01, p=.049, decreasing as a function of the Distance factor (contiguous: 32.81%; non-contiguous 1-apart: 14.15%; non-contiguous 2-apart: 7.64%). The pairwise comparisons for digit strings showed significant differences in the transposition costs between contiguous and non-contiguous manipulations: contiguous vs. non-contiguous 1-apart, F1(1,31) = 34.32, p<.001; F2(1,71) = 49.93, p<.001 (21.88% vs. 5.56%); contiguous vs. non-contiguous 2-apart, F1(1,31) = 42.11, p<.001; F2(1,71) = 61.95, p<.001 (21.88% vs. 2%). However the difference in the magnitude of the transposition cost between non-contiguous 1-apart and 2-apart manipulations was not significant, F1(1,31) = 2.86, p=.1; F2(1,71) = 1.51, p=.22 (5.56% vs. 2%). The pairwise comparisons for symbol strings showed a very similar pattern to that observed for digit strings: contiguous vs. non-contiguous 1-apart: F1(1,31) = 34.52, p<.001; F2(1,71) = 13.85, p<.001 (15.97% vs. 5.21%); contiguous vs. non-contiguous 2-apart: F1(1,31) = 34.87, p<.001; F2(1,71) = 12.21, p=.001 (15.97% vs. 5.30%); non-contiguous 1-apart vs. non-contiguous 2-apart: F1(1,31) < .001, p=.96; F2(1,71) = 0.02, p=.87 (5.21% vs. 5.30%). Summing up, there is a clear gradation of the magnitudes of the transposition costs for letter strings depending on the distance effect. Regarding digit and symbol strings, the transposition cost was larger for contiguous manipulations than for the two non-contiguous manipulations that did not differ from each other.

### 2.2 The Type of Character Effect:

Subsequent pairwise comparisons for contiguous manipulations showed that the transposition costs were significantly different between the three types of characters: letters vs. digits, F1(1,31) = 12.64, p=.001; F2(1,71) = 12.95, p=.001 (32.81% vs. 21.88%); letters vs. symbols, F1(1,31) = 30.23, p<.001; F2(1,71) = 31.88, p<.001 (32.81% vs. 15.97%); digits vs. symbols, F1(1,31) = 4.79, p=.036; F2(1,71) = 4.96, p=.029 (21.88% vs. 15.97%). Pairwise comparisons for non-contiguous 1-apart manipulations showed significant differences in the transposition costs between letter and digit strings, F1(1,31) = 9.15, p=.005; F2(1,71) = 16.19, p<.001 (14.15% vs. 5.56%), and between letter and symbol strings, F1(1,31) = 14.95, p=.001; F2(1,71) = 12.22, p=.001 (14.15% vs. 5.21%). However, the difference in the transposition cost between digit and symbol strings was not significant, F1(1,31) = 0.03, p=.85; F2(1,71) = 0.01, p=.9 (5.56% vs. 5.21%). The pairwise comparisons for non-contiguous 2-apart manipulations only showed significant differences in the transposition costs between letter and digit strings, F1(1,31) = 4.53, p=.041; F2(1,71) = 4.23, p=.043 (7.64% vs. 2%). The other comparisons were not significant (all ps > .13). In a nutshell, we found there is a clear gradation of the Type of Character effect for the contiguous manipulations, showing the largest cost for letter strings followed by digit strings and finally by symbol strings. Regarding the Type of Character effect in the non-contiguous 1-apart manipulations, the transposition cost was larger for letter strings than for digit and symbol strings (which did not differ from each other).

## Discussion

The present study employed a perceptual matching task in order to investigate participants’ ability to judge that two strings of items are different when the difference lies in the transposition of two characters compared with the substitution of two characters. In line with prior research, we found that detecting a transposition change was harder than detecting a substitution change, an effect referred to as a transposition cost [25]. We also investigated whether this transposition cost was modulated as a function of the distance between the characters involved in the change (contiguous, non-contiguous with one intervening character, and non-contiguous with two intervening characters). In line with prior research using masked priming [5, 7, 29, and 30], we found evidence for a decrease in transposition costs as distance increased.

Most important, however, is that we investigated whether such transposition costs, and their modulation by transposition distance, would be the same for the different types of stimuli we tested, or differ as a function of stimulus type. According to one account of how letter position information is encoded during visual word recognition [10,11], transposed-letter effects are driven by generic positional noise that operates identically for different types of familiar visual stimuli. A very different account of letter position encoding postulates that transposed letter effects are not only driven by positional noise, but also by the flexibility that is inherent in the very mechanism that codes for positional information [15,16,18]. According to the latter approach to letter position coding we should find evidence for letter-specific effects in the present study, thus providing a replication and extension of the prior evidence in this direction [25].

**Figure 3:**
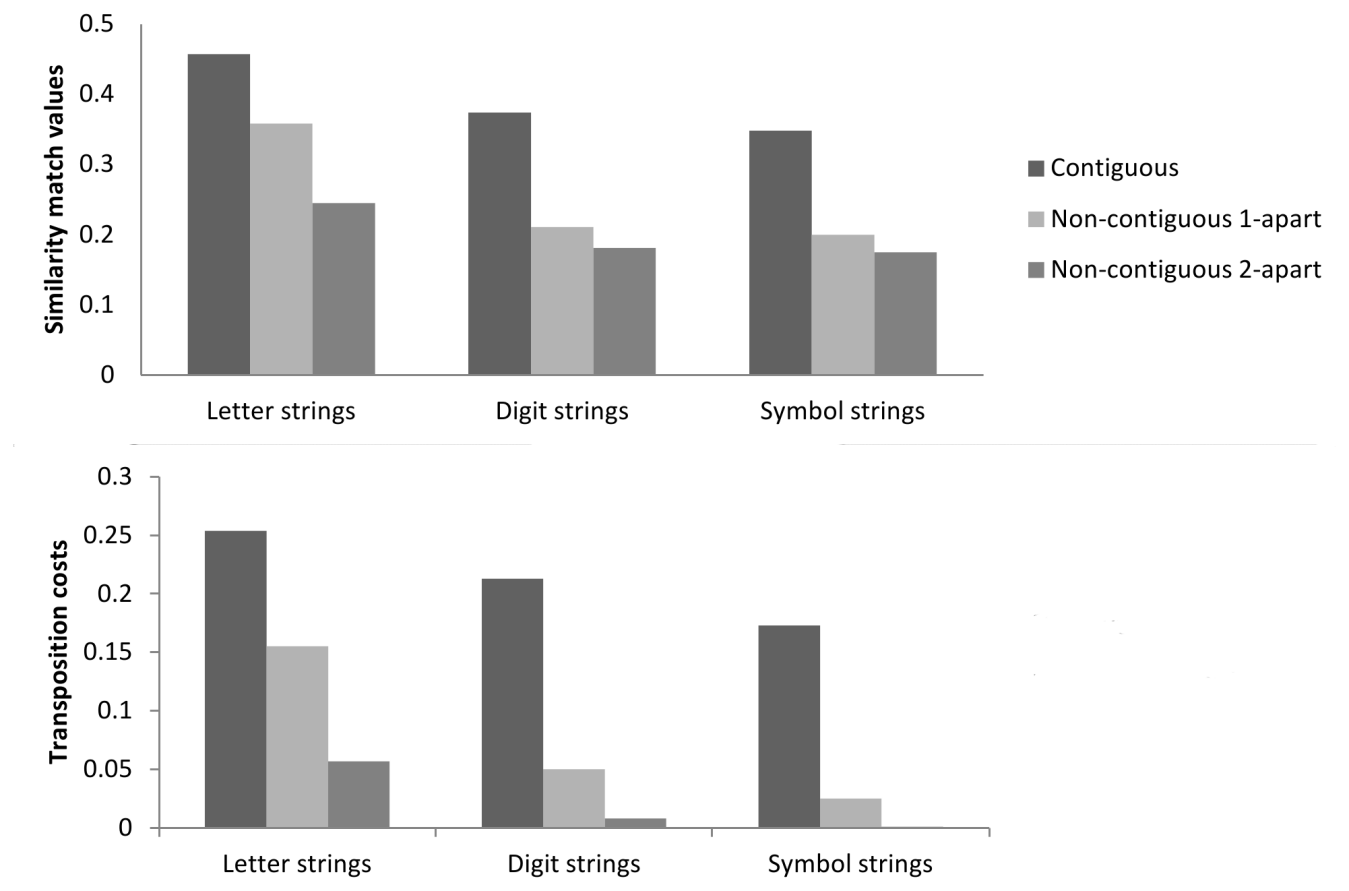
Fits of the overlap model. Top panel: similarity match values from the overlap model for the letter, digit and symbol strings for the different experimental conditions (note that different values of the *s*-parameter were used to simulate different effects for different types of character). Bottom panel: transposition cost values from the overlap model for the three types of string obtained by subtracting match values for letter transpositions from match values for letter replacements for each type of letter transposition.

The present data show that transposition costs are larger for contiguous character transpositions than for non-contiguous character transpositions for all types of materials. The overall graded effects of contiguity are in line with the predictions of the Overlap model [10]. This model considers that objects’ locations in a sequence are modeled over position which occurs before the distinction of object types. In that sense, the probability of a given character being at a given position diminishes as function of the distance from its exact location following a Gaussian distribution. In the case of transposed-character manipulations, the overlap model predicts that transposing contiguous characters would lead to a higher perceptual overlap with regard to the reference (namely, a larger cost) than transposing non-contiguous characters involving one intervening character, which in turn will lead to greater perceptual overlap than transposing non-contiguous characters involving two intervening characters. The results of simulations with the Overlap model are shown in Figure 3. According to this point of view, transposition effects are a consequence of object position uncertainty as depicted by general models of visual attention [34,35]. This process of position encoding is assumed to be effective regardless of the type of visual objects. Thus, this apparent flexibility in the positional information encoding would be a by-product of a general property of the visual recognition system.

However and more importantly, our results also revealed that transposition costs were overall larger for letter strings than for both digit and symbol strings. Furthermore, the distance factor had a different impact on these transposition costs for letter stimuli compared with digit and symbol stimuli. More precisely, letter stimuli showed a gradual decrease in transposition cost with increasing distance, whereas digit and symbol stimuli both showed a decrease in transposition cost from contiguous to non-contiguous transpositions, but no significant effect of the number of intervening characters in the non-contiguous conditions. Considering similar parameter estimations for all types of characters, these present data cannot be accommodated by the Overlap model since it does not a priori predict any interaction between transposition effects and the type of character. However, this model would be able to account for the differences in the transposition costs between letter, digit and symbol strings by tuning the values of the *s*-parameter (which corresponds to the standard deviations of the letter distribution function) as a function of the type of input (letter, digits or symbols; see Figure 3) in order to fit the data. Nonetheless, it is worth mentioning that even with this parameter tuning, the model would run into difficulties to fit the high error rate found for contiguous letter transpositions. Hence, even if the Overlap model seems a reasonably good candidate to account for most of the data here reported, the pieces of data that are not readily captured by the model seem to favor models of orthographic processing based on letter-specific principles (over and above domain-general principles).

**Figure 4:**
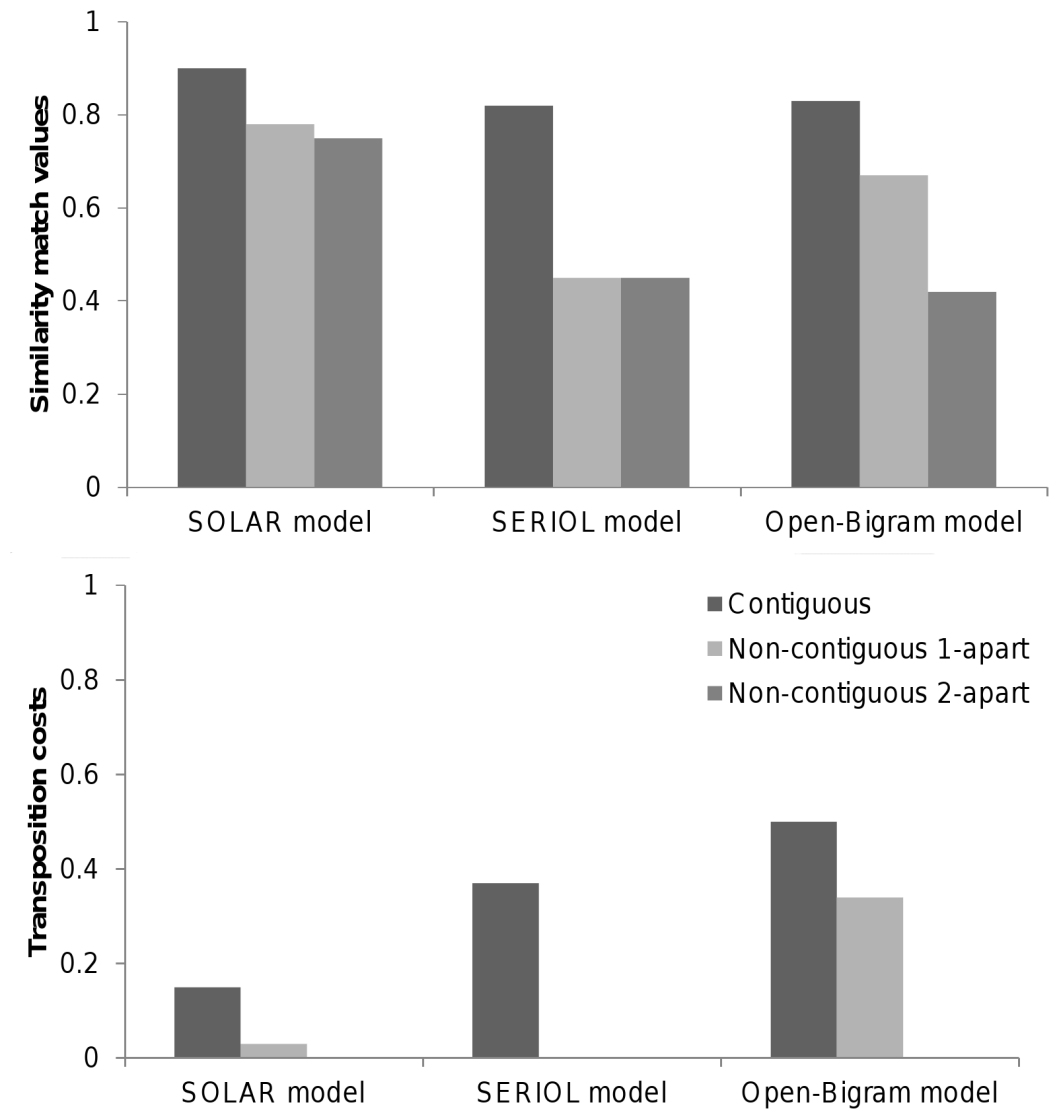
Fits of the SOLAR, SERIOL and Open bigram model. Top panel: similarity match values for the SOLAR, SERIOL and Open bigram models for the different types of transposition. Bottom panel: transposition cost values for the three models obtained by subtracting match values for letter transpositions from match values for letter replacements for each type of letter transposition.

Greater transposition costs for letters compared with other kinds of familiar visual stimuli, is a natural consequence of models that code for letter-in-string position in a flexible manner. This is the case for models that employ open-bigram coding [16,18] and spatial coding [15]. Such flexibility in position coding is used in order to achieve a location-invariant (i.e., independent of viewing position) sublexical representation of orthographic information that codes for position-in-word rather than position-on-retina. However, current implementations of these models would appear to not generate the amount of flexibility required to capture the transposition costs that occurred with 2-character separations. The results of simulations (The match scores were obtained from the MatchCalculator application (v. 1.9) developed by Colin Davis. This application is available at: http://www.pc.rhul.ac.uk/staff/c.davis/Utilities/MatchCalc/index.htm) are shown in Figure 4. These simulations revealed that none of the models predicted a transposition cost when the change involved letters separated by two letters. It should nevertheless be noted that a more recent version of open-bigram coding [17] has opted for increased flexibility by implementing distance as a parameter that can change as a function of encoding conditions.

Most important, however, is that all these simulations are noise-free. Now, assuming that noise operates on position coding mechanisms, whatever their nature, then we have a simple means to extend the above models in order to capture the complete pattern of results observed in the present study. Here we provide one example of this extension, couched in the framework of Grainger and van Heuven’s (2003) model of orthographic processing. In this particular model, generic positional noise operates at the level of retinotopic letter detectors [20], and this noise will affect coding of word-centered bigram representations. Assuming minimal positional noise here such that a letter at position N can be erroneously encoded as being at positions N-1 or N+1, then computation of a bigram with distance 2 can sometimes lead to computation of a bigram formed of two letters separated by four letters (distance 4). Noisy retinotopic coding increases the flexibility of word-centered open-bigram coding, enabling such models to capture TL effects with two intervening letters. This is very different from the so-called “overlap open-bigram model” (see 20 according to which positional uncertainty only arises at the level of retinotopic letter detectors, and it is due to this positional noise that non-contiguous bigrams are encoded [36].

In sum, the observed differences in transposed-character effects found for letters compared with both digits and symbols in the present study, points to the existence of letter-specific position-coding mechanism. Generic positional noise operating on an otherwise rigid position coding mechanism cannot capture the present results. Nevertheless, generic positional noise must still influence the processing of letter stimuli, just like any other kind of visual stimulus, and it might be the case that when added to existing flexible position-coding mechanisms, this would provide the additional flexibility required to provide a complete account of the present data.
